# Guttural Pouch Mycosis: A Three-Step Therapeutic Approach

**DOI:** 10.3390/vetsci11010041

**Published:** 2024-01-19

**Authors:** Olivier M. Lepage

**Affiliations:** Centre for Equine Health, National Veterinary School of Lyon, VetAgro Sup, University of Lyon, 69280 Lyon, France; olivier.lepage@vetagro-sup.fr

**Keywords:** guttural pouch, endoscopy, trans-arterial coil embolization, topical oxygen therapy, common carotid artery ligation, equine, epistaxis

## Abstract

**Simple Summary:**

Clinical signs of guttural pouch mycosis in equids tend to be nonspecific but can include epistaxis, cranial nerve disorders, uni- or bilateral mucoid nasal discharge or a combination of these symptoms. On the basis of these warning symptoms, the equine practitioner will confirm the diagnosis through an endoscopy and must be prepared, sometimes in an emergency, to propose the most appropriate therapeutic approach, depending on the clinical situation. A three-step therapeutic approach to this condition is described, including temporary common carotid artery ligature at the barn, the trans-arterial coil embolization procedure in a specialized equine surgery center to stop or prevent epistaxis, and topical oxygen therapy to improve local defences in the respiratory mucosa of the guttural pouches while reducing the development of the mycotic organism.

**Abstract:**

The temporary ligation of the common carotid artery is performed as an emergency aid in cases of guttural pouch mycosis. Its usefulness is put into perspective after an anatomical summary of arterial vascularization involving a guttural pouch. It helps to better understand the need for the cranial (cerebral) and caudal (cardiac) occlusion of an arterial rupture by embolization in order to achieve maximum success in preventing and treating an hemorrhage. Topical oxygen therapy used alone or in a multimodal approach with embolization surgery is performed to promote healing of the inflammatory and mycotic lesions observed when an individual is affected. In conclusion, this three-step therapeutic approach should enable the equine practitioner to better orient their decision tree when faced with this condition which, while rare, can be potentially fatal if poorly treated.

## 1. Introduction

Lesions associated with guttural pouch mycosis (GPM) can be observed in any part of both compartments of one or both guttural pouches (GP), but are typically found on the internal carotid artery (ICA), along the dorsal roof of the medial compartment. It is still unclear why specific anatomical areas are invaded and what factors predispose them to the development [1] of a variety of fungal organisms [2,3].

**Figure 1:**
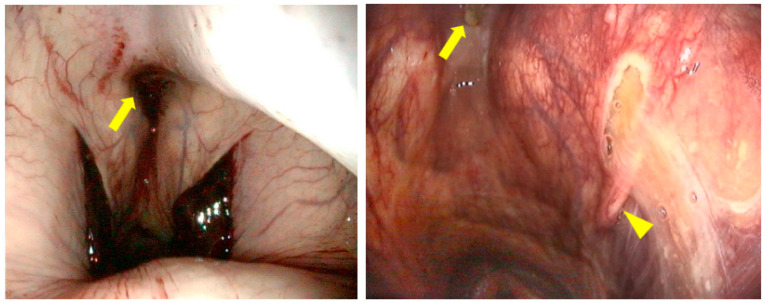
Endoscopic aspect of the pharynx with: (**left**) the presence of clots at both pharyngeal orifices and bleeding coming from a fistula in the retropharyngeal space (arrow); (**right**) the presence of secretion from fistulas in the retropharyngeal space (arrow) and in the wall of the left pharyngeal orifice (arrowhead).

A presumptive diagnosis of GPM must be made when the history or the initial examination by the veterinarian reveals symptoms of a cranial nerve deficit, an unusual nasal discharge and/or epistaxis. An endoscopic examination of the GP is then recommended in order to obtain a definitive diagnosis. The veterinarian in charge of this examination is expected to have a good knowledge of the pharyngeal structure and function during the endoscopic examination of the pharynx and the GP [4,5,6,7], and to recognize and locate any alterations. This examination starts with a good assessment of the pharynx followed by both GPs. The general appearance of the pharynx should be examined to discover whether there is a reduction in air passage through a collapse of the pharyngeal roof, the presence of one or more fistulas in its wall (Figure 1), laryngeal hemiparesis, or signs of dysphagia, combined or not with a permanent or intermittent dorsal displacement of the soft palate. All these observations are of interest in order to establish a further treatment plan [8] and prognosis. Endoscopic examination of the GP is performed only in the absence of fresh blood or a clot at one or both GP pharyngeal orifices.

If epistaxis is suspected, based or not on history, it is recommended that endoscopy of the pharynx is limited and to evaluate if the bleeding comes from one or both GPs by observing the pharyngeal orifices. Epistaxis is suspected when the person bringing the animal reports the presence of blood drying at the entrance of one or both nasal cavities, or the presence of blood on the floor of where the horse lives in the two weeks preceding the consultation. Limiting the endoscopic examination of the pharynx is a useful precaution when the surgical team is not ready for a possible arterial occlusion [9]. Even in cases where an experienced surgical team is ready, it is preferable to go straight to an embolization surgery, so as not to compromise it by making it impossible for the anesthetists to induce the horse for general anesthesia [9] or to keep him sedated for standing surgery [10] following the onset of an induced major arterial hemorrhage.

If an accurate identification is impossible during the endoscopic examination because entering was not justified, or due to landmarks obscuring by blood or clots, all arteries in the affected GP should be occluded by the surgeon. This involves occluding the maxillary artery, the ICA and the external carotid artery (ECA). Once the diagnosis has been clarified, there are three main steps in the management of a GPM. Step 1 involves the emergency management of the case during the initial examination at the barn, step 2 is performed in a specialized equine surgery center to prevent fatal hemorrhage by performing definitive arterial occlusion, and step 3 is intended to support the animal and resolve the various symptoms and deficits.

## 2. Materials and Methods

### 2.1. Anatomical Structures of Interest

**Figure 2:**
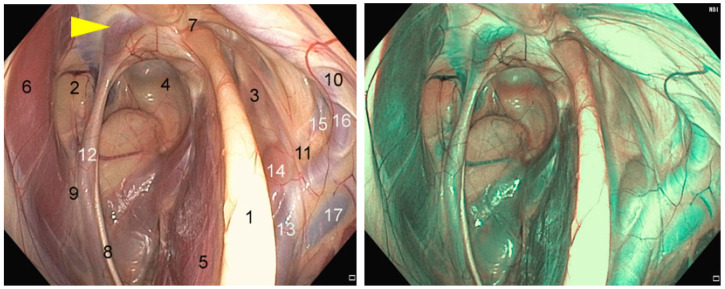
Endoscopic anatomy of a left guttural pouch in an adult horse observed with: (**left**) no optical filter; (**right**) narrow band imaging (NBI) technology using an optical filter to contrast between blood vessels and the surrounding tissue, increasing visibility of vascular structures. (1) Stylohyoid bone; (2) medial compartment; (3) lateral compartment; (4) atlanto–occipital joint; (5) stylohyoid m.; (6) longus capitis m.; (7) temporohyoid joint; (8) CNXII, hypoglossal n.; (9) CNXI, accessory n.; (10) CNVII, facial n.; (11) carotid plexus; (12) internal carotid a.; (13) external carotid a.; (14) caudal auricular a.; (15) superficial temporal a.; (16) maxillary a.; (17) maxillary v.; arrowhead, sigmoid flexure of the ICA.

The ICA is coursing around the caudal aspect of the medial compartment wall for the majority of its length, delivering blood from the heart to the brain [6]. This artery can be identified as being the first major branch of the common carotid artery (CCA) at its trifurcation, giving rise also to the external carotid artery (ECA) and the occipital artery. [5] The ICA forms, at the entrance to the cranial cavity, a sigmoid flexure (Figure 2), connecting, with the opposite ICA via a transverse branch, the caudal intercarotid artery (*a. intercarotica caudalis*). It then gives rise to the caudal communicating artery (*a. communicans caudalis*) and ends in the middle cerebral artery (*a. cerebri media*) and the rostral cerebral artery (*a. cerebri rostralis*). The union of these arterial branches with the opposite side forms the cerebral arterial circle (*circulus arteriosus cerebri*) or circle of Willis [11]. The occipital artery gives rise to a caudal branch which anastomoses with the vertebral artery (*a. vertebralis*). The left and right vertebral arteries then join with the ventral spinal artery (*a. spinalis ventralis*) to form the basilar artery (*a. basilaris*), which continues rostrally to join the cerebral arterial circle via the caudal communicating artery. If there is no direct contact with the GP, the occipital artery, through its communication with the cerebral arterial circle, participates with the ICA in retrograde blood flow in the event of hemorrhage from a rupture of the ICA [12].

After giving rise to the ICA and the occipital artery, the CCA continues as a large trunk, the ECA. As it approaches the ventral surface of the medial compartment of the GP, it gives rise to the linguofacial artery (*a. linguofacialis*). The ECA then continues its course dorsally in the mucosa of the lateral compartment, giving rise to the caudal auricular artery (*a. auricularis caudalis*) and the superficial temporal artery (*a. temporalis superficialis*), in order to become the maxillary artery (*a. maxillaris*). The maxillary artery travels in the mucous membrane of the roof of the lateral compartment in a cranial direction. During its course in the alar canal, it gives rise to the external ophthalmic artery (*a. ophtalmica externa*), which in turn produces the central retinal artery (a. centralis retinae) and the external ethmoidal artery (*a. ethmoidalis externa*), certain branches of which join the cerebral arterial circle. On leaving the alar canal, the maxillary artery is extended by the palatine artery (*a. palatina major*), which runs along the lingual surface of the teeth to anastomose with the artery on the opposite side behind the upper incisors [11].

The numerous collateral arteries (mandibular alveolar artery, ethmoidal and external ophthalmic arteries, infraorbital artery and major palatine artery) may be responsible for retrograde arterial flow at the level of a lesion of the ECA [13] or the maxillary artery, preventing the formation of a thrombus. Even if the ECA is more rarely affected by mycosis of the GP, the surgical approach to an alteration of this artery must always consider the fact that, as with the ICA, retrograde blood flow must be blocked in addition to the normograde blood flow from the heart [14].

### 2.2. Step 1: Common Carotid Artery Temporary Ligature

Unilateral [14,15] or bilateral [16] temporary ligation of the CCA is performed on the horse to reduce epistaxis. Most cases have unilateral GPM and unilateral bleeding, but in cases of severe bleeding from one GP, epistaxis may be bilateral and the side for ligation of the CCA should then be based on history and on the highest amount of blood [14]. In some cases, the owner is present when the hemorrhage starts, or they have observed dried blood in a nostril in the days prior to the initial consultation, which indicates to the veterinarian which side should be ligated.

**Figure 3:**
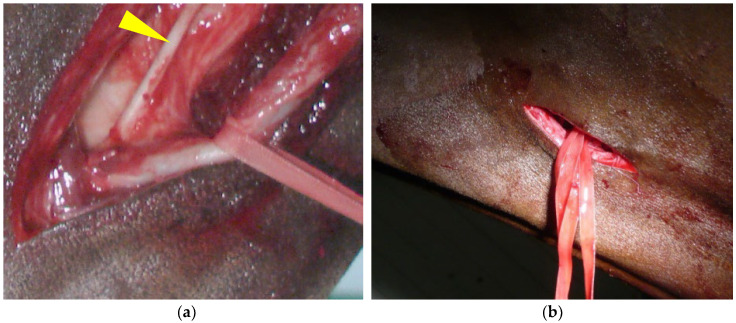
Common carotid artery (CCA) temporary ligature: (**a**) the CCA sheath has been incised with dissector forceps and umbilical tape has been placed around the artery. The vagosympathetic trunk (arrowhead) is observed deeper in the surgical wound. (**b**) The 10-cm skin incision made at the junction of the proximal and middle-third of the neck, just above the jugular vein, will be left open to heal by second intention after the removal of the temporary ligation.

In order to perform this ligation, the veterinarian needs to position himself comfortably facing the jugular groove [15]. An approximative 10-cm skin incision is made at the junction of the proximal and middle-third of the neck, just above the jugular vein (Figure 3). The fibers of the brachiocephalicus muscle and, deeper, the omohyoid muscle are bluntly separated. The carotid trunk is identified with the tip of a finger and elevated by blunt dissection. The use of 21-cm dissector forceps is very useful at this stage. The CCA sheath is incised with the forceps, and the vagosympathetic trunk is carefully separated from the CCA and replaced in the incision (Figure 3a). The CCA continues to be elevated from the incision with the dissector forceps and an ombilical tape or a retraction tape (surgical loop 4 mm; B. Braun S.A) or a large (minimum USP 5—decimal 8) braided non-absorbable (polyester, Ethibond^®^) or absorbable (polyglactin 910, Vicryl^®^) suture material, placed around the artery. One surgical knot for the ligature and the use of a large diameter suture material reduces trauma to the arterial wall and makes the removal of the temporary ligature easier. Some veterinarians prefer to use a Penrose for this manipulation.

Subsequent primary closure of the wound often causes surgical site infection and wound dehiscence [14]. It is recommended that the surgical incision is not closed and to cover the area with a neck bandage, which should be changed daily. If no other treatments are planned for economic reasons or lack of qualified personnel, the healing process will not exceed 3 weeks. If an embolization surgery [8,9] is planned, this facilitates access to the CCA for the surgeon who will perform the procedure after the removal of the temporary ligature.

At this stage, before arterial occlusion surgery is performed, unless the patient is in a state of severe shock, it is not advisable to give a blood transfusion or to put the patient on fluid therapy, as this could unnecessarily increase blood pressure and encourage further hemorrhaging.

### 2.3. Step 2: Trans-Arterial Coil Embolization (TACE)

Once a horse arrives at a specialized equine surgery center, it is always advisable to treat the condition as a matter of emergency. It is impossible to predict when a new hemorrhagic episode may occur. The basic principle of surgical treatment is to embolize on the cranial (cerebral) and caudal (cardiac) sides of the arterial rupture, whatever its location, so as to prevent normo-grade and retrograde arterial blood from flowing through the breach in the wall of the artery involved. The most precise technique with the highest success rate is TACE [8,9] under the guidance of a fluoroscope. The technique can be performed despite active bleeding, allowing for the identification and correct occlusion of all sources of the hemorrhage.

**Figure 4:**
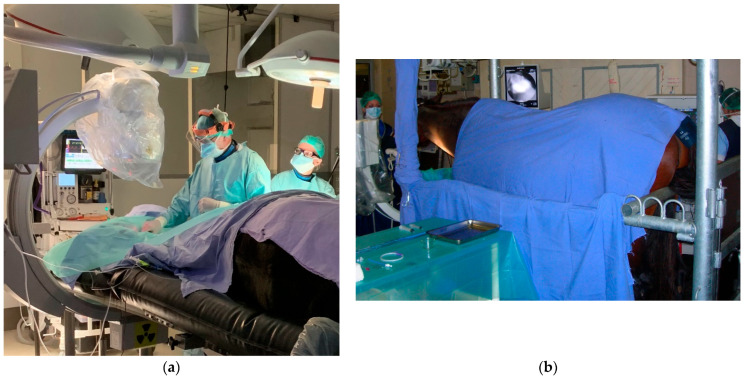
Trans-arterial coil embolization procedure performed: (**a**) under general anesthesia; (**b**) in a standing horse with an assistant at the front of the horse to hold the head and neck straight and the anesthetist positioned at the tail. The use of radiation protection clothing and equipment by all staff in the operating theater should be noted.

The TACE procedure is usually performed under general anesthesia [9] but it is also feasible in a standing horse [10] (Figure 4). However, this minimally invasive method for arterial occlusion in a standing horse should be reserved for surgeons experienced with the technique performed under general anesthesia and to clinical cases with major neurologic deficits or very high-risk parameters for general anesthesia, because sudden collapse during the procedure has been reported [17].

After routine induction of anesthesia, the horse is placed in lateral recumbency, with the affected GP uppermost [9]. The head and neck are positioned on a Plexiglas table, in order to enable fluoroscopy. It is extremely rare for two arteries in two GPs to be ruptured at the same time. If this is the case, it is necessary to flip the horse from one side to the other side on the table or perform a standing surgery. An area over the jugular groove at the junction of the upper and middle-third of the neck is prepared aseptically. In cases where temporary CCA ligation has been performed [14,15], the suture material eventually applied to the surgical incision is removed prior to surgical site preparation.

**Figure 5:**
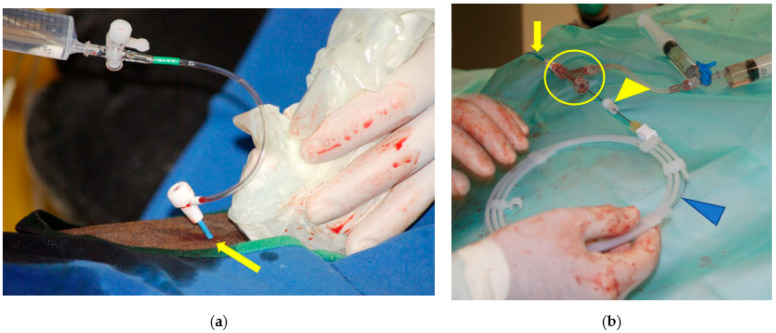
Percutaneous trans-arterial coil embolization procedure under ultrasound guidance in a recumbent horse under general anesthesia: (**a**) placement of a 6F-introducer system into the CCA (arrow); (**b**) a 6F single end-hole nylon angiographic catheter (arrow) has been advanced cranially into the introducer. On the opposite site, a coil (yellow arrowhead) is pushed with a guide-wire (blue arrowhead) through a Y-adapter (Tuohy-borst side-arm, Cook) (yellow circle).

The CCA is isolated, as described in step 1 of this three-step therapeutic approach, and punctured to place a 6F-introducer system. The use of a suture material less elastic (braided polyester or polyglactin 910 or a retraction tape) than a Penrose gives greater stability during the placement of the introducer. A 6F single end-hole nylon angiographic catheter is then advanced cranially into the CCA under fluoroscopic guidance. Angiography and angiographic catheter placement can also be safely performed using a percutaneous approach to the CCA under ultrasound guidance (Figure 5) in standing or anesthetized horses [18]. The approach requires the presence of an ultrasound scanner in the surgery room, and hematoma formation can impair the procedure. This technique is not recommended for surgeons with little experience with the TACE procedure or if there is an urgent need to embolize due to active bleeding, as the percutaneous approach to the CCA is more time-consuming than the open approach.

**Figure 6:**
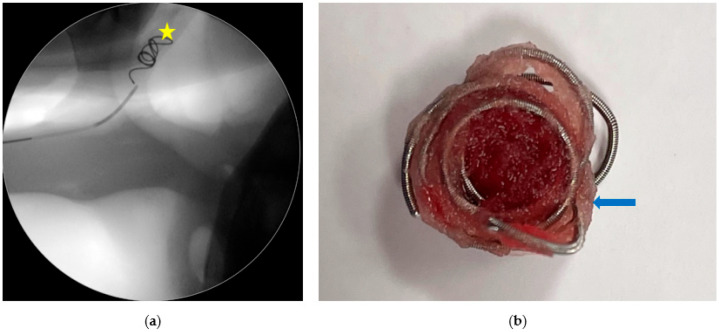
Trans-arterial coil embolization procedure under fluoroscopic guidance: (**a**) a first coil (star) is placed into the internal carotid artery on the caudal (cardiac) side of the rupture; (**b**) appearance of a coil soaked briefly in blood to highlight the occlusion rapidly induced by the dacron-fibers (arrow) covering a stainless-steel occluding spring embolization coil.

Multiple angiograms are performed by hand-injection of iohexol bolus (Omnipaque, GE Healthcare SAS, Buc, France) in order to identify normal arterial structures, alterations to these structures and any variability in the anatomy [19]. Once the planned site of occlusion is reached, the diameter of the artery is estimated in order to select dacron fiber-covered, stainless steel occluding spring embolization coil of a proper diameter (Figure 6). Additional smaller imbricating embolization coils are then introduced until complete occlusion is obtained. Both angiographic and introducer catheters are withdrawn at the end of the procedure and the CCA is sutured at the site of introducer sheath removal. The artery puncture is primarily closed with a taper-point 5-0 (decimal 1) or smaller non-absorbable monofilament (silk, nylon or polypropylene). The surgical incision is routinely sutured to ensure first intention wound healing or left to heal by second intention if a temporary ligation of the CCA has been performed prior to the embolization surgery. In this case, the site of the surgery was covered with a neck bandage, to be changed daily for the first 5–7 days before leaving to air.

It is only when the surgeon has completed the necessary embolization that he informs the anesthetist that he can start more extensive fluid therapy or even a blood transfusion, if the animal’s condition requires it.

### 2.4. Step 3: Topical Oxygen Therapy (TOT)

**Figure 7:**
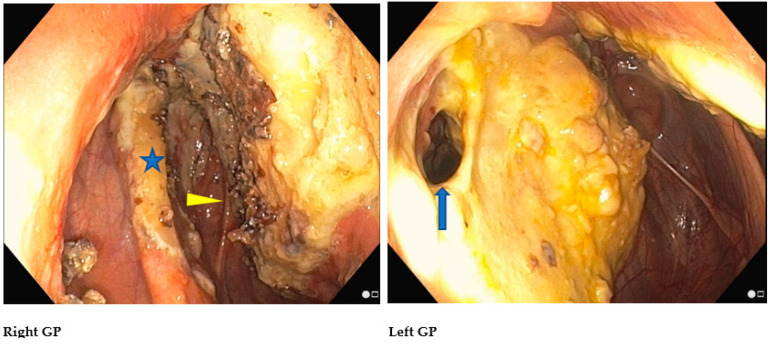
Bilateral GPM in an adult horse with signs of dysphagia showing macroscopic inflammatory lesions: (**right GP**) of the stylohyoid bone (arrowhead) and the medial compartment involving CNXII-hypoglossal n. (arrow); (**left GP**) the thin membrane separating it from the right GP shows a fistule (arrow).

If the management of bleeding originating from the GP has a high success rate with the TACE procedure [8], the resolution of the macroscopic inflammatory lesions in cases of GPM is highly variable (Figure 7) and the resolution of a neurologic deficit is inconstant and challenging. Topical oxygen therapy intends to safely modulate GPM by reversing the course of the disease. Based on the encouraging results of TOT in 14 horses (8 experimental horses, 6 clinical cases) [20], the technique is recommended: (1) alone if no history of epistaxis exists, and lesions are not overlying major arteries; and (2) in a multimodal approach with a TACE procedure if the patient is at risk of epistaxis.

**Figure 8:**
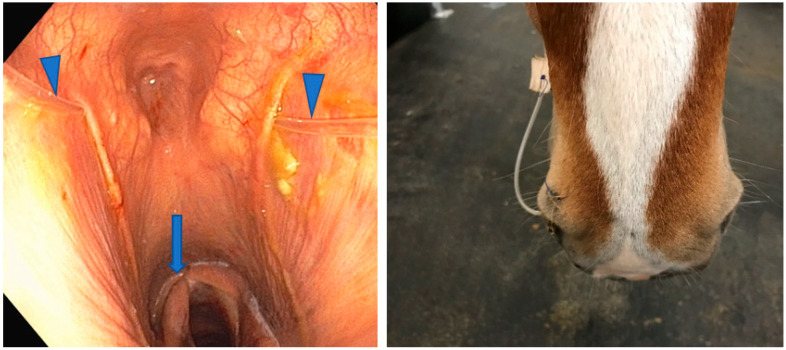
Topical oxygen therapy. **Left** image: commercial 8 Fr 135 cm long GP polyurethane catheter (arrowhead) is placed in both guttural pouches of a horse bilaterally affected; note the right laryngeal hemiparesis (arrow). **Right** image: securing the catheter to the right nostril of a horse treated unilaterally.

In order to realize TOT, an upper respiratory tract endoscopy is mandatory, with the endoscope placed in the contralateral nasal passage of the GP to be treated in order to enable the observation of the placement of a commercial 8 Fr 135 cm long GP polyurethane catheter (MILA International Inc., Florence, KY, USA). This catheter is used for oxygen administration and is left in place for as long as needed (Figure 8). It is replaced only if it is dislodged from the GP to be treated. The most common reason for changing is displacement caused by horses rubbing their noses and destroying the catheter fixation. For this reason, apart from meals and TOT sessions, attaching a grazing muzzle to the halter is recommended [20]. The outside part of the GP catheter is secured to the halter and connected via its Luer lock extremity to an extender connected to a bottle of oxygen or to a wall outlet of a master oxygen system [20]. Water is not used to humidify the oxygen.

**Figure 9:**
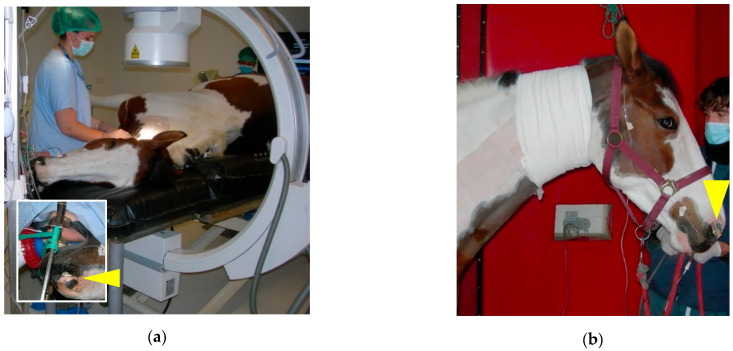
Topical oxygen therapy: (**a**) under general anesthesia during a TACE procedure; and (**b**) on a standing animal with extension of the GP catheter connected to a wall outlet of a master oxygen system. GP catheter (arrow).

Topical oxygen therapy sessions can be performed under general anesthesia during a TACE procedure and/or on a standing animal (Figure 9). In both situations, TOT should be administered three to four times a day at 15 L/min for periods of 30 to 60 min, depending on animal temperament, and a 5 to 7 days period is usually sufficient to reverse the course of the disease. Horses are sedated only if necessary.

**Figure 10:**
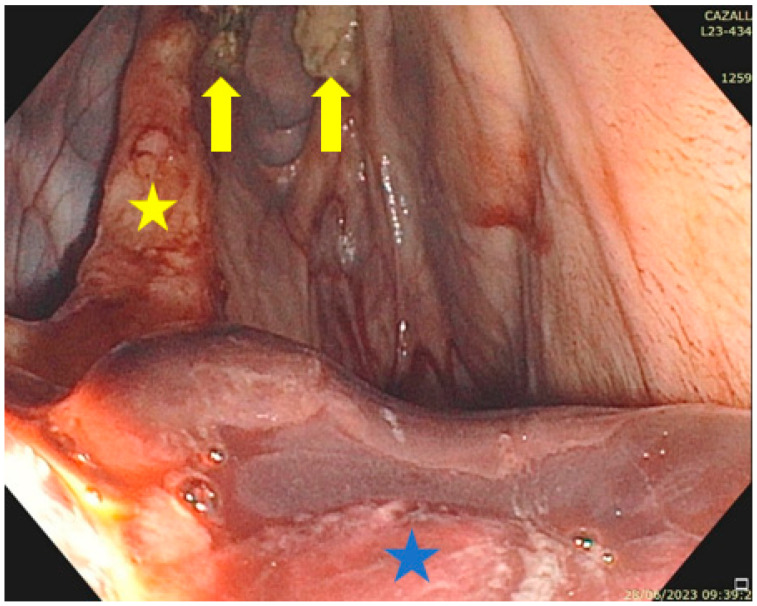
Mycosis of a right GP three days after a TACE procedure. A large blood clot is seen on the floor of the medial compartment (blue star), indicating that TOT can now be started because the mycotic lesions (arrows) seen on the roof of this compartment are no longer in contact with the blood; stylohyoid bone (yellow star).

For the oxygen to be effective, the TOT procedure must not be started if the GP is full of blood. The practitioner needs to wait, sometimes 2 to 5 days after a TACE procedure, until a clot has formed that is no longer in contact with the mycotic lesions (Figure 10).

Oxygen administration for TOT is easy to perform in a standing horse with no adverse effects. After two administrations, macroscopic inflammatory lesions decrease more quickly in size in the treated GP, nasal discharge resolves progressively and partial or total recovery of a neurologic deficit (2/4 laryngeal hemiparesis, 3/5 dysphagia, 1/2 dorsal displacement of the soft palate, and 1/1 Horner’s syndrome) is reported [20].

## 3. Discussion

When a diagnosis of GPM is made, it is essential to ascertain the extent of the lesions and whether there is neurologic deficit. The prognosis is poorer when such deficits are present at the time of the initial clinical examination. The presence of hemorrhage from a GP does not necessarily mean that a mycosis is present [12]. Other causes include the rupture of an aneurysm, especially if the hemorrhage is substantial [17], bacterial infection, [21] a rupture of the *longus capitis* [22] and/or *rectus capitis ventralis* [23] muscles, and a fracture of the stylohyoid bone [24]. If a hemorrhage is active or in remission, a temporary ligature must be made or predisposed around the CCA on the side of the GP that seems most at risk. However, it is important to be aware that this ligation is not always effective, and bleeding could quickly recur or continue. Short-term continuous clinical follow-up (5 days) of ten resting horses that had undergone permanent ligation of the CCA revealed no neurologic deficits or impaired vision. On the other hand, second intention healing of the surgical site causes less wound infection than first intention closures [14]. During temporary bilateral occlusion of the CCA, no neurological or visual deficits have been reported either [16]. This is explained by the fact that the cerebral arterial circle is sufficiently irrigated by the spinal artery, avoiding any cerebral ischemia.

A technique under general anesthesia for ligating the ICA can also be performed [25]. This ligation shows some effectiveness in preventing fatal hemorrhage, since six out of thirty individuals showed mild to severe episodes of epistaxis during the first four weeks after ligation, and fatal epistaxis was recorded only once [26]. This surgery requires general anesthesia, given the difficulty of access. Recurrence of hemorrhage is always possible through retrograde arterial flow. Owners should be warned that with permanent ligation of the CCA or the ICA, fatal hemorrhage may occur at any time up to 6 weeks after ligation.

A technique combining the ligation of the ICA and the placement of a balloon catheter is then performed [27] and a few years later, the same team proposed a technique of ECA ligation and catheterization to eliminate retrograde arterial flow from arteries proximal to the caudal alar foramen [28]. More recently, balloon catheter occlusions of the ICA, ECA and maxillary artery have been performed in standing horses [29].

These techniques should always be considered in the absence of a fluoroscope, but are less precise compared to TACE, as they do not allow for the assessment of the lesion site or the identification of anatomical variations. Complications are mainly due to catheter misplacement or infection of the surgical site [30]. But cerebral embolisms, responsible for paraplegia and originating from a thrombus of the ICA, have also been described [26], as has the development of blindness on the same side as the arterial ligations [31]. Loss of vision is seen after recovery from general anesthesia, and it is suggested that it is a lack of blood supply to the optic nerve and retina that causes the ischemia responsible for blindness. Blood to the eye comes from the internal and external ophthalmic arteries, which branch off from the ICA and ECA, respectively.

Ideally, if there are no economic constraints, the horse or, less frequently, donkey, with a suspected GPM should be transferred to a specialized equine surgery center equipped with a fluoroscope and with a surgeon familiar with the TACE procedure [8,9]. This technique provides a safe, rapid and effective method for various arterial occlusions. Among the factors contributing to the greater success of preventing a fatal hemorrhage, we have (1) the early diagnosis and referral of the case, (2) an experienced surgeon utilizing the TACE procedure, (3) a stock of specific instruments with a wide size range of coils, and (4) the proper positioning of the patient to prevent any movements during surgery [17]. The TACE technique is constantly evolving to improve its approach [32] and to incorporate new technologies such as a specific artery closure device (Angio-Seal VIP, Terumo, Leuven, Belgium) to seal the puncture wound in the CCA after the removal of the introducer catheter. [33]

Many topical and systemic treatments have been tested in attempts to overcome mycosis in the GP, often with little success [26,34,35]. It is also very difficult to assess the efficacy of any treatment, since it has been found that spontaneous resolution is observed both experimentally [36] and after a TACE procedure, where the complete resolutions of mycosis lesions are recorded in the weeks or months following surgery, without any additional treatment [17].

**Figure 11:**
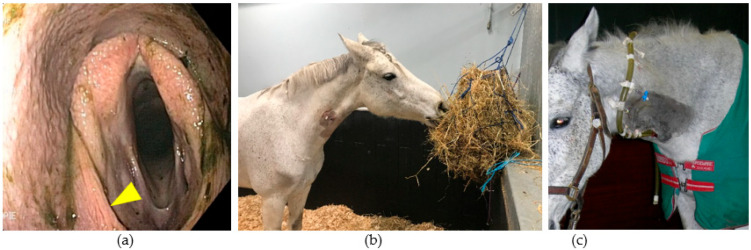
Dysphagia consecutive to a GPM: (**a**) appearance of the pharynx at endoscopic examination showing food particles on all mucous membranes, left laryngeal hemiparesis and dorsal displacement of the soft palate (arrow); (**b**) adapting feed intake to reduce induced coughing; and (**c**) esophagostomy to reduce the risk of aspiration pneumonia and provide the necessary energy to the body.

In some horses affected by a GPM, the persistence of a neurologic deficit such as dysphagia (Figure 11) may be responsible for a poor vital prognosis and prompts us, in step 3 of the described approach, to adapt an individualized support treatment to the animal, such as nutritional intake via an esophagostomy. This situation also requires us to find a solution to reduce local inflammation and provide an environment less conducive to the development of fungal organisms, even if it is not clear that this is the primary causative agent. For example, a mare presented for epistaxis, diagnosed with an aneurysm of the right ICA and treated with a TACE procedure, was later (2 months) diagnosed with GPM but without any clinical signs [20].

Salpingopharyngeal fistulation in horses with GPM aims to create a direct static opening into the GP from the pharynx in order to cause a regression of fungal plaques due to a change in the environment [37]. This technique is not appropriate for horses that are at risk of bleeding and it is difficult to assess the effect of the surgery, given that various other treatments were administered at the same time.

Another way of modifying the environment of a GP is to administer oxygen. Given the positive experience with oxygen therapy in the resolution of human fungal diseases [38] and its reported use in horses [39,40], a two-phase study to assess TOT for the treatment of GPM in horses was performed [20]. In adult horses, TOT alone or in a multimodal approach with a TACE procedure is feasible and safe, and has a propensity to reverse the course and the progression of macroscopic inflammatory lesions without additional lavage, topical or systemic treatments. Trans-endoscopic surgical debridement is not necessary either, and if it is carried out, it is very important not to create any lesions in the respiratory mucosa of the GP, given the wealth of vascular and nerve structures in the region.

## 4. Conclusions

Guttural pouch mycosis remains a mystery. On the other hand, the risks of permanent neurological deficit or fatal hemorrhage are present at all times, and we cannot wait for the response to a spontaneous regression or to a hypothetical medical treatment before acting. The three-step therapeutic approach to manage equids with a GPM described herein should enable the practitioner to temporarily stop or reduce a hemorrhage, if present, with a CCA ligation, to definitively rule out this problem as a referral for a TACE procedure, and to propose a complementary TOT to modify the GP environment. Oxygen therapy will help reverse the course of the disease, most probably by affecting the development of the fungi and increasing the effectiveness of the body’s natural defenses.

## Data Availability

Data are contained within the communication.

## References

[1] Lepage O., Perron M.-F., Cadoré J.-L. (2004). The mystery of fungal infection in the guttural pouches. Vet. J..

[2] Cook W.R., Campbell R.S., Dawson C. (1968). The pathology and aetiology of guttural pouch mycosis in the horse. Vet. Rec..

[3] Ludwig A., Gatineau S., Reynnaud M.C., Cadoré J.L., Bourdoiseau G. (2004). Fungal isolation and identification in 21 cases of guttural pouch mycosis in horses (1998–2002). Vet. J..

[4] Piat P., Cadoré J.-L. (2023). Endoscopic Anatomy of the Equine Guttural Pouch: An Anatomic Observational Study. Vet. Sci..

[5] Lepage O., Orsini A., Grenager N.S., de Lahunta A. (2022). Guttural pouch disease. Comparative Veterinary Anatomy—A Clinical Approach.

[6] Baptiste K.E., Naylor J.M., Bailey J., Barber E.M., Post K., Thornhill J. (2000). A function for guttural pouches in the horse. Nature.

[7] Deluzurieux M., Desjardins I., Nolf M., Guidi E., Depecker M., Cadoré J.-L. (2013). Endoscopic analysis of guttural pouch opening in horses. J. Exp. Appl. Anim. Sci..

[8] Lepage O.M., Piccot-Crezollet C. (2005). Transarterial coil embolization in 31 horses (1999–2002) with guttural pouch mycosis: A two years’ follow-up. Equine Vet. J..

[9] Léveillé R., Hardy J., Robertson J.T., Willis M.A., Beard W.L., Weisbrode S.E., Lepage O.M. (2000). Transarterial occlusion of the internal and external carotid and maxillary arteries for prevention of hemorrhage from guttural pouch mycosis in horses. Vet. Surg..

[10] Benredouane K., Lepage O.M. (2012). Trans-arterial coil embolization of the internal carotid artery in standing horses. Vet. Surg..

[11] Ghoshal N.G., Nanda B.S. (1975). Heart and arteries. Sisson and Grossman’s the Anatomy of the Domestic Animals.

[12] Lepage O.M. (1994). Hémorragie dans les poches gutturales. 1. Anatomie, diagnostic et étiologie. Prat. Vet. Eq..

[13] Freeman D.E., White N., Moore J. (1990). Guttural pouch mycosis. Current Practice of Equine Surgery.

[14] Lepage O.M. (1995). Hémorragie dans les poches gutturales. 2. Traitements, pronostic et complications. Prat. Vet. Eq..

[15] Profizi C., Lepage O.M. (2014). Réaliser une ligature de l’artère carotide commune en urgence. Prat. Vét. Eq..

[16] Wyn-Jones G., Jones R.S., Church S. (1986). Temporary bilateral carotid artery occlusion as an aid to nasal surgery in the horse. Equine Vet. J..

[17] Lepage O.M. (2015). Challenges associated with the diagnosis and management of guttural pouch epistaxis in equids. Equine Vet. Educ..

[18] Maninchedda U., Lepage O.M., Gangl M., Benredouane K. (2015). Percutaneous ultrasound-guided arterial angiography for trans-arterial coil placement in anesthetized and standing horses. Vet. Surg..

[19] Bonilla A.G., Scansen B.A., Hurcombe S.D., Mudge M.C. (2015). Potential for iatrogenic coil embolization of the caudal cerebellar artery during treatment of internal carotid artery bifurcatipon in two horses with gutturalpouch mycosis. J. Am. Vet. Med. Assoc..

[20] Lepage O.M., Di Francesco P., Moulin N., Gangl M., Texier G., Marchi J., Cadoré J.-L. (2021). The effect of topical oxygen therapy in horses affected with mycosis of the guttural pouch: An experimental pilot study and a case series. Animals.

[21] Smith K.M., Barber S.M. (1984). Guttural pouch hemorrhage associated with lesions of the maxillary artery in two horses. Can. Vet. J..

[22] Sweeney C.R., Freeman D.E., Sweeney R.W., Rubin J.L., Maxson A.D. (1993). Hemorrhage into the guttural pouch (auditory tube diverticulum) associated with rupture of the longus capitis muscle in three horses. J. Am. Vet. Med. Assoc..

[23] Knight A.P. (1977). Dysphagia resulting from unilateral rupture of the rectus capitis ventralis muscles in a horse. J. Am. Vet. Med. Assoc..

[24] Nation P.N. (1978). Epistaxis of guttural pouch origin in horses: Pathology of three cases. Can. Vet. J..

[25] McIlwraith C.W. (1987). Extra diverticular ligation of the internal carotid artery for guttural pouch mycosis. Equine Surgery Advanced Techniques.

[26] Greet T.R.C. (1987). Outcome of treatment in 35 cases of guttural pouch mycosis. Equine Vet. J..

[27] Freeman D.E., Donawick W.J. (1980). Occlusion of internal carotid artery in the horse by means of a balloon tipped catheter: Clinical use of a method to prevent epistaxis caused by guttural pouch mycosis. J. Am. Vet. Med. Assoc..

[28] Freeman D.E., Ross M.W., Donawick W.J., Hamir A.N. (1989). Occlusion of the external carotid and maxillary arteries in the horse to prevent hemorrhage from guttural pouch mycosis. Vet. Surg..

[29] Genton M., Farfan M., Tesson C., Laclaire A.-L., Rossignol F., Mespoulhes-Rivière C. (2021). Balloon catheter occlusion of the maxillary, internal, and external carotid arteries in standing horses. Vet. Surg..

[30] Jennings A., Lepage O.M., Mair T., Sherlock C. (2019). Surgical site infection after occlusion of the internal carotid artery with a thrombectomy catheter: Five cases. Equine vet. Educ..

[31] Hardy J., Robertson J.T., Wilkie D.A. (1990). Ischemic optic neuropathy and blindness after arterial occlusion for treatment of guttural pouch mycosis in two horses. J. Am. Vet. Med. Assoc..

[32] Munoz J., Iglesias M., Lloret Chao E., Bussy C. (2015). Ultrasound guided transarterial coil placement in the internal and external carotid artery in horses. Vet. Surg..

[33] Arantza V., Laborda A., Serrano-Casorran C., Fuente S., Romero A., Vazquez F.J. (2022). Percutaneous ultrasound guided carotid access and puncture closure with angio-seal in horses. Animals.

[34] Speirs V.C., Harrison I.W., Veenendaal J.C., Baumgartner T., Josseck H.H., Reutter H. (1995). Is specific antifungal therapy necessary for the treatment of guttural pouch mycosis in horses?. Equine Vet. J..

[35] Cousty M., Tricaud C., De Beauregard T., Picandet V., Bizon-Mercier C., Tessier C. (2016). Ligation of the ipsilateral common carotid artery and topical treatment for the prevention of epistaxis from guttural pouch mycosis in horses. Vet. Rec..

[36] Greppi M.C., Guillot J., Melloul E., Bourdoiseau G., Lepage O., Cadoré J.-L. (2016). Experimental induction of mycotic plaques in the guttural pouches of horses. Med. Mycol..

[37] Watkins A.R., Parente E.J. (2018). Salpingopharyngeal fistula as a treatment for guttural pouch mycosis in seven horses. Equine Vet. J..

[38] García-Covarrubias L., Barratt D.M., Bartlett R., Metzinger S., Van Meter K. (2002). Invasive aspergillosis treated with adjunctive hyperbaric oxygenation: A retrospective clinical series at a single institution. South. Med. J..

[39] Dobesova O., Schwarz B., Velde K., Jahn P., Zert Z., Bezdekova B. (2012). Guttural pouch mycosis in horses: A retrospective study of 28 cases. Vet. Rec..

[40] Di Francesco P., Lepage O.M., Moulin N., Cadoré J.-L. Gli effetti e l’utilizzo dell’ossigeno iperbarico nell’Aspergillosi delle tasche gutturali del cavallo: Studio preliminare. Proceedings of the 22nd SIVE International Congress.

